# Investigation of histopathological and radiological effects of surfactant treatment in an experimental female rat model of lung contusion

**DOI:** 10.22038/ijbms.2019.32357.8258

**Published:** 2019-10

**Authors:** Yasin Keskin, Cihan Bedel, Nesrin Gökben Beceren

**Affiliations:** 1 Beyhekim State Hospital, Department of Emergency Medicine, Konya, Turkey; 2 Health Science University Antalya Training and Research Hospital, Department of Emergency Medicine, Antalya, Turkey; 3 Süleyman Demirel University Faculty of Medicine, Department of Emergency Medicine, Isparta, Turkey

**Keywords:** Blunt chest trauma, Chest trauma, Contusion, Lung contusion, Rat, Surfactant

## Abstract

**Objective(s):**

Pulmonary contusion (PC) is a clinical entity that often accompanies blunt traumas. We aimed to investigate the radiological and histopathological effects of surfactant treatment in an experimental rat model in which lung contusion was formed by blunt thoracic trauma.

**Materials and Methods:**

50 female Sprague-Dawley rats were used. Five groups were formed randomly. In groups 2, 4, and 5 lung contusion was made by the drop-weight method after anesthesia. Intratracheal surfactant was administered in the 4th hr in groups 3 and 4 and in the 24th hr in groups 4 and 5. All rats were sacrificed and their lungs removed at 48 hr after contusion. Alveolar edema, congestion, hemorrhage, destruction, leukocyte infiltration, immune staining were examined histopathologically.

**Results:**

When the first thoracic CT scans were evaluated, we observed two rats with rib fractures and four rats with pneumothorax. 4 and 48 hr thoracic CT evaluation contusion and atelectasis showed no statistically significant decrease (*P*>0.05). After sacrifice of group 2, in macroscopic evaluation, there was a heterogeneous contusion and hemorrhagic appearance in the lungs of rats and less hemorrhagic appearance was observed in Groups 4 and 5 than in Group 2. In comparison of Immunohistopathological findings, surfactant treatment showed a statistically significant decrease in leukocyte infiltration scores (*P*=0.046). Immunohistopathologically, surfactant group had more staining but only statistically significant when compared to groups 4 and sham. (*P*=0.036).

**Conclusion:**

Surfactant treatment may be of significant benefit in lung contusion secondary to blunt chest trauma, and further prospective evidence of its efficacy in such disorders is needed.

## Introduction

Pulmonary contusion (PC) is a common problem that is the most frequently diagnosed intrathoracic injury. It can affect approximately 17%–25% of adult patients (1). It may lead to pneumonia, acute lung injury, and adult respiratory failure syndrome. The pathophysiology of PC includes inflammation, increased alveolocapillary permeability, pulmonary edema, ventilation/perfusion mismatching, increased intrapulmonary shunting, and a loss of compliance (2). 

Surfactant covering the surface of the lung alveoli is released from type II pneumocytes. Surfactant, a complex of lipids and proteins, prevents the alveoli from collapsing expiration. In the absence of surfactant or in the presence of a low level of surfactant, the lung injury is aggravated by various mechanisms including increased atelectasis, inflammation, and deterioration of epithelial integrity (3, 4).

Our hypothesis in this study is that 100 mg/kg dose surfactant treatment significantly benefits in lung contusion in rats and to evaluate the histopathological effects of the surfactant in lung tissue.

## Materials and Methods

***Experimental animals***

This study was performed in the Animal Research Laboratories and was approved by the Ethics Committee for Animal Experiments. Fifty female Sprague–Dawley rats weighing 180– 265 g and aged 4–6 months were enrolled. All rats were acclimatized for at least a week before the operation to allow them to adjust to the laboratory environment. Throughout the experiment, animals received the standard rat diet and water. They were housed individually in polycarbonate microisolator cages in a controlled environment with a temperature of 20–26 ^°^C. They were exposed to a light-dark (L/D) cycle of 24 hr (L/D = 12/12 hr). The animals were cared for according to the guidelines set by the Guide for Care and Use of Laboratory Animals.

***Surgical procedure***

**Figure 1:**
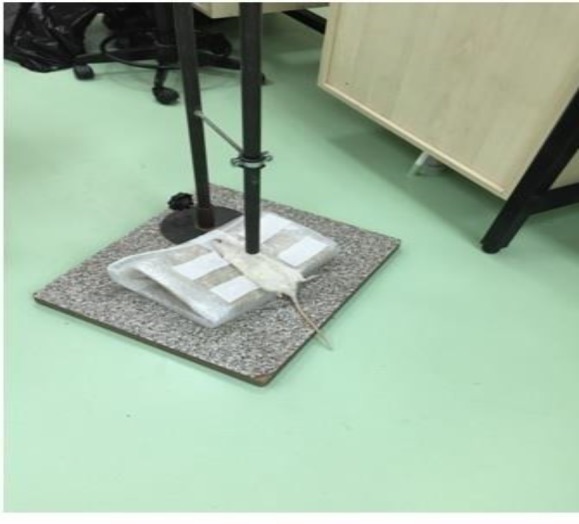
The apparatus used for the high weight drop method is shown. After anesthesia, the rat was laid on the metal plate, and another metal free roller is placed on the chest part, and a 200 g metal cylinder was lowered from a height of 100 cm to the chest area of the rats. The procedure was performed once per rat

Rats were randomly divided into five equal groups (n=10). Anesthesia was administered intraperitoneally with ketamine HCl (Ketalar®, Eczacibasi, Istanbul Turkey) 50 mg/kg + xylazine (Rompun®, Bayer, Istanbul Turkey) 10 mg/kg. During the process, rats were asleep so that their respiration would continue spontaneously. The rat model for bilateral PC was induced by high-fall energy (1). The experimental device is illustrated in Figure 1. Using a device, 200 g metal cylinder was lowered from a height of 100 cm to the chest area of ​​the rats. Impact energy (E) was calculated using E = mgh, where m is the mass (kg), g is the gravitational acceleration (9.8 m/sec), and h is the height where the weight is left (m). The frictional force was ignored. Blunt thoracic trauma by 1.96-J force was applied to the rats.

***Groups***

*Group 1:* (sham) After anesthesia, thorax computed tomography (CT) and lung samples were taken in this group.

*Group 2: *(contusion control) contusion of lungs was created under anesthesia and pre and post-contusion lung samples were taken. Thorax CT was performed at the 48th hr.

*Group 3:* (surfactant control) Intratracheally 100 mg/kg/ dose surfactant was administered before the contusion model was formed. Thorax CT was performed at 0th and 48th hr. Lung samples were taken.

*Group 4:* (early period contusion treatment group, early 4th hr) Intratracheally 100 mg/kg/ dose surfactant was administered at 4th hr before the contusion model was formed. Thorax CT was performed at 0-4th and 48th hr. Lung samples were taken.

*Group 5:* (late period contusion treatment group, early 24th hr) Intratracheally 100 mg/kg/ dose surfactant was administered at 24th hr before the contusion model was formed. Thorax CT was performed at 0-4th and 48th hrs. Lung samples were taken.

**Figure 2:**
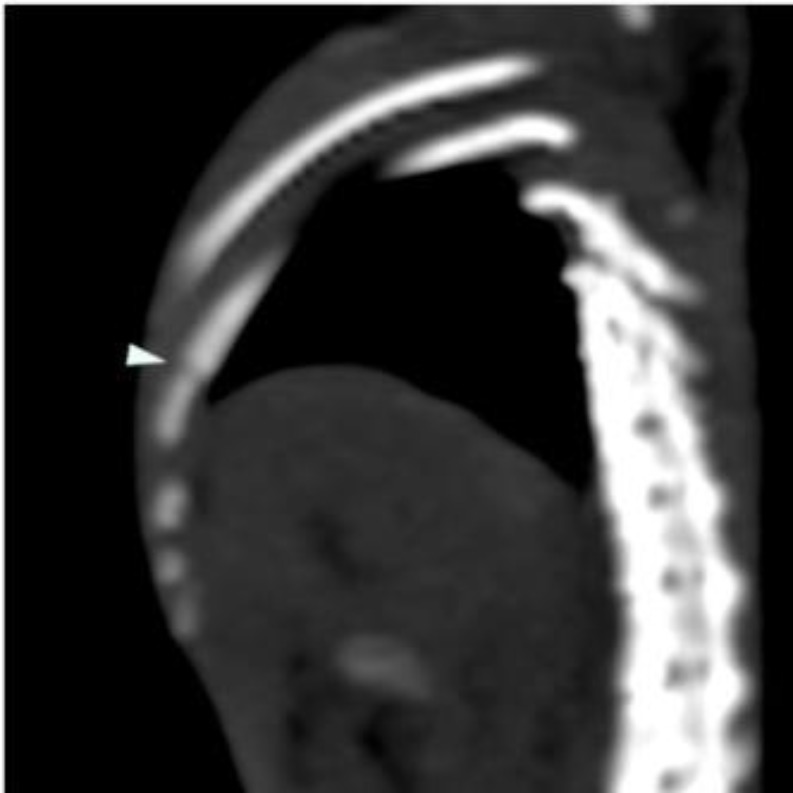
In thorax CT the rib fracture is indicated by the white arrow

**Figure 3:**
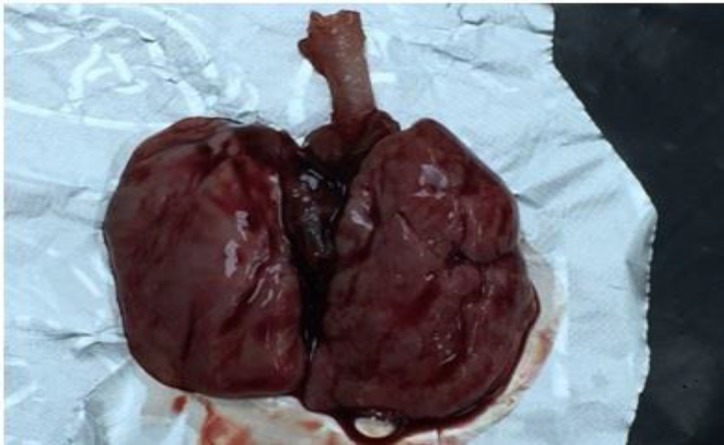
After sacrification macroscopic hemorrhagic appearance of the lung tissue obtained from the control group of the lung contusion

**Figure 4:**
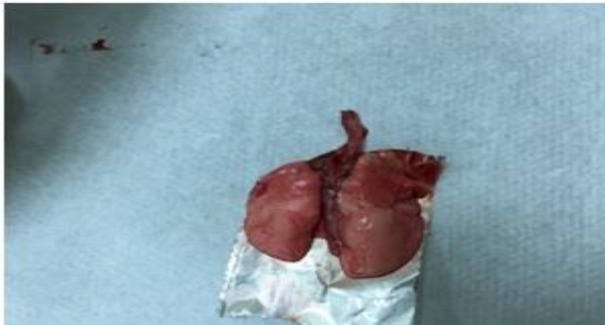
Macroscopic appearance of lung tissue obtained after sacrification of the contusion treatment group

**Figure 5:**
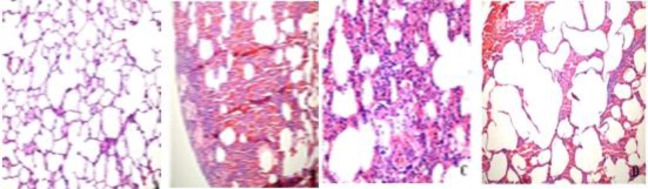
(A): Normal lung tissue without contusion (HE x 200) (B): Hemorrhagic and edematous appearance in lung tissue with contusion (HE X100) (C): Leukocytic infiltration appearance of lung tissue with contusion (HE X400) (D): Alveolar disruption appearance of lung tissue with contusion (HE X100)

**Table 1:** Statistical comparison of histopathologic results of groups

**Histopathological parameters**	**Group 1**	**Group 2**	**Group 3**	**Group 4**	**Group 5**	***P***
**Alveolar hemorrhage**	0.77±0.66	1.88±0.64	2.14±0.9	2.43±0.78	2.57±0.53	**<0.001**
**Alveolar edema / congestion**	1.11±0.78	1.01±0.1	1.57±0.53	1.57±0.78	1.71±0.78	**0.020**
**Alveolar distruption**	0.89±0.33	1.25±0.71	1±0.57	0.57±0.53	1.14±0.37	0.265
**Leukocytic sequestration**	1.44±0.72	2.5±0.53	2.14±0.69	2.14±0.69	1.71±0.75	0.405
**Immun staining**	1.33±0.71	1.63±0.74	1.86±0.69	2±0.57	1.86±0.69	0.364

Rats with agenesis, pleural effusion, and pneumonic infiltration, which were detected radiologically in the first CT of the rats were excluded from the study. The animals underwent median sternotomy and sacrificed. After weighing the wet weights of the rats’ lungs, histopathological and immunohistochemical studies were taken in containers filled with 10% formaldehyde and examined double blinded by a pathologist. Sections were taken from these tissues and stained with Hematoxylin and Eosin (HE). A total of 4 rats died after anesthesia.

Histopathological changes (intra alveolar hemorrhage, alveolar edema, congestion, alveolar disruption, and leukocytic infiltration) were studied. Alveolar edema/congestion, alveolar hemorrhage, disruption, and immune staining were scored from 0 to 3: 0 = no pathology, 1 = mild (<10%), 2 = moderate (10-25%), and 3 = severe (25%). Leukocytic infiltration was used to demonstrate the severity of inflammation. Each section was divided into ten sub-sections, and leukocyte infiltration was scored from 0 to 3 in each sub-section with HE x 400 magnification. 0 = no leukocytes, 1 = ≤10 leukocytes, 2 = 10-45 leukocytes, and 3 = ≥ 45 leukocytes. The averages of the numbers obtained for comparison were obtained.

***Statistical analysis***

The obtained data were evaluated using SPSS 18.0 (SPSS Inc., Chicago, Illinois, USA) statistical package program for Windows. Kruskal-Wallis variance analysis and one way ANOVA methods were used. A value of *P*<0.05 was considered statistically significant.

## Results

***a-Radiological evaluation***

In our study, when the first thoracic CT scans were evaluated, two rats with rib fractures and four rats with pneumothorax were observed (Figure 2). Atelectasis was observed in 8 rats. In the 4th and 48th hr thoracic CT evaluation, there were no statistically significant decreases in contusion and atelectasis (*P*>0.05).

***b-Macroscopic findings***

During the post-contusion observation, after the contusion, it was observed that there was an acute respiratory depression in the rat, but they regained normal respiratory movements within seconds. After sacrification of group 2 in macroscopic evaluation, there was a heterogeneous contusion and hemorrhagic appearance in the lungs of rats. Less hemorrhagic appearance was observed in groups 4 and 5 than in group 2 (Figures 3 and 4).

***c- Immunohistopathological findings***

The comparison of the results between the groups was performed using the scoring system designed for alveolar hemorrhage, congestion/edema, alveolar disruption, and leukocytic infiltration. It was seen that contusion caused lung damage and leukocytic sequestration in terms of all parameters. But surfactant treatment showed no statistically significant decrease in leukocyte infiltration scores (*P*>0.05). When the groups were compared in terms of alveolar hemorrhage and congestion/edema, there was a significant difference compared to the control group (*P*<0.001; *P*=0.02, respectively) (Figures 5a-d) (Table 1).

Immunohistopathologically, group 2 was less stained than the sham group. When we compare the surfactant group to control and sham groups, the surfactant group had more staining but only statistically significant when compared to group 4 and the sham group (*P*=0.036).

## Discussion

Thoracic trauma accounts for 10–15% of all traumata, and the mortality rate is 20–25%. Extra tension and tear in the alveoli, separation of the alveoli from the bronchioles, intra-alveolar hemorrhage, interstitial edema, and alveolocapillary damage after the trauma are defined as PC. Pulmonary congestion occurs in 30–75% of major thorax trauma, which is a serious injury with high mortality and morbidity rates (5, 6).

Radiologically, there is widespread infiltration and patch-like consolidation in the contusion. The main concern in the treatment of these patients is respiratory support, depending on the clinical and laboratory findings. The radiological appearance of the lung contusion begins to resolve within a few days (48–72 hr) with appropriate treatment (7, 8).In our study as a result of radiological imaging at 4th and 48th hrs, there were no statistically significant decreases in contusion and atelectasis.

In a recent study, the amount of surfactant from bronchoalveolar lavage made in the contusion-generated rats was gradually decreased and reached the lowest level at 24 hr (9). In our study, acute lung contusion resulting from blunt thoracic trauma, surfactant was used for treatment, and thoracic CTs were used to demonstrate damage to the contusion radiographically. Lung tissue was evaluated histopathologically. Thus, we aimed to demonstrate the ability of surfactant to suppress inflammatory mechanism, to increase antioxidant activity, and to be used for epithelial damage, which is important in decreasing mortality and morbidity in lung contusion.

In one study, the effects of exogenous pulmonary surfactant were investigated by generating acute lung injury in rats during severe burns. Exogenous pulmonary surfactant has been shown to improve oxygenation and alleviate membrane permeability of pulmonary capillaries in pulmonary edema and burn (10). In our study, alveolar edema and disruption were more beneficial compared with the control group in patients with isolated blunt thoracic trauma.

Raghavendran *et al.* indicated lymphocyte dominance and intra alveolar edema were detected in the histopathologic evaluation of rats treated with a lung contusion. It has also been shown that the use of surfactant reduces the severity of contusion in the lung, reduces inflammatory reaction, and may have protective effects (9). In another study, it has been shown that surfactant can increase lung function effectively after unilateral lung contusion is applied (11). In our study, similar findings were found, and leukocyte infiltration was statistically significant compared to the sham group (*P*=0.046).

In a study, it was found that hemorrhage and edema were more frequent in the alveoli in the early post-traumatic period, but leukocytic infiltration and atelectasis were prominent in the alveolar space at 24 hr after trauma (12). In another study, with the loss of surfactant and increased alveolar surface tension, significant reductions in pulmonary compliance, atelectasis, and stability problems have been identified (13). Our study had similar radiological and histopathological results, but we believe that more experimental animals and parameters will give more meaningful results. 

In our study, alveolar hemorrhage was increased in the sham and control groups compared to surfactant groups. Alveolar hemorrhage is present as a side effect of surfactant therapy (14, 15). Our result can be attributed to the side effect of the drug, but the low number of rats in the study has been a restrictive situation. 

In our study, antioxidant and blood gas values ​​were not studied in rats, which is a restrictive condition. We think that further studies, which may show the improvement of some inflammatory and oxidative parameters after the administration of surfactant in lung-contusion rats, as well as the improvement of the parameters of lung physiology, will give more healthy information about the activity of the surfactant.

## Conclusion

100 mg/kg/dose surfactant treatment significantly benefited in lung contusion in rats secondary to blunt chest trauma, and histopathologically ensured the recovery of the lung tissue. 

## Conflicts of Interest

The authors declare no conflicts of interest relevant to this study.
